# A Hybrid RF Coil for Whole‐Brain Imaging in Non‐Human Primates at 7 T

**DOI:** 10.1002/mrm.70361

**Published:** 2026-03-24

**Authors:** Elias Djaballah, Éric Giacomini, Paul‐François Gapais, Michel Luong, Alexis Amadon, Qi Zhu

**Affiliations:** ^1^ Cognitive Neuroimaging Unit, INSERM, CEA Université Paris‐Saclay, NeuroSpin Center GIF‐SUR‐YVETTE France; ^2^ BAOBAB Université Paris‐Saclay, CEA/Joliot/NeuroSpin GIF‐SUR‐YVETTE France; ^3^ Université Paris‐Saclay, CEA/DRF/IRFU/DACM GIF‐SUR‐YVETTE France

**Keywords:** dipole, macaque, RF coil, transmit array, ultra‐high‐field

## Abstract

**Purpose:**

Ultra‐high field MRI (≥ 7 T) offers unprecedented potential for mapping non‐human primate (NHP) brain function. However, complex electromagnetic systems are required to meet the challenges of UHF imaging and the mechanical demands of awake NHP studies. To address these challenges, we developed a hybrid RF coil system optimized for whole‐brain fMRI of macaques at 7 T.

**Methods:**

The hybrid coil system combines a 6‐channel preamplifier‐decoupled transceive dipole array with a 16‐channel loop receive array, housed in a compact structure designed for awake imaging that maintains open visual fields. Key design features include compatibility with sphinx‐position monkey chairs and head fixation systems required for awake experiments. Electromagnetic simulations guided dipole design to optimize uniform transmit performance and deep penetration. Phantom and in vivo experiments validated these predictions using anesthetized macaques and evaluated the system's readiness for awake imaging.

**Results:**

The hybrid coil demonstrated uniform $B_{1}^{+}$ distribution with phase‐shimmed transmission across the brain. It supported robust parallel imaging, enabling up to *R* = 3 × 2 acceleration factors for high‐resolution acquisitions. The achieved whole‐brain tSNR in fMRI is comparable to that of an existing 8‐TX/24‐RX coil designed for imaging anesthetized macaques. Critically, submillimeter (0.75 mm isotropic) resting‐state fMRI revealed clear default‐mode network connectivity, confirming the system's capability for high‐quality functional imaging.

**Conclusion:**

By effectively addressing both the technical challenges of ultra‐high field MRI and the mechanical constraints associated with visual stimulation in awake NHPs, this hybrid coil system provides a powerful tool for advancing our understanding of primate brain function.

## 1 Introduction

The development of high‐performance coils is critical in advancing ultra‐high field (UHF) magnetic resonance imaging (MRI), particularly for applications involving non‐human primates (NHPs). At 7 Tesla (7 T) and beyond, UHF MRI systems offer significant advantages in spatial resolution and sensitivity, enabling researchers to capture fine‐grained anatomical and functional details that underlie cognitive processes. However, these high magnetic fields introduce significant electromagnetic challenges that require innovative RF coil designs tailored not only for the complexities of UHF MRI [1, 2, 3, 4, 5, 6], but also for the unique demands of NHP imaging.

Compared to lower field strengths, UHFs present several unique issues. First, the shorter wavelengths of RF signals exacerbate standing wave effects, leading to inhomogeneous $B_{1}^{+}$ field during transmission. This results in uneven excitation angle patterns and spatially variable image contrast [7] and, in some cases, even complete signal loss [8]. Additionally, $B_{0}$ field inhomogeneities and susceptibility artifacts become more pronounced, causing distortions and signal loss, particularly at air‐tissue interfaces. Elevated specific absorption rate (SAR) further limits imaging protocols, while physiological noise, such as breathing induced motion artifacts, becomes more significant.

Beyond these general UHF challenges, NHP brain imaging has its own unique difficulties due to anatomical differences and experimental requirements. Macaque brains are approximately 10 times smaller in weight, and their neocortex is about 25% thinner [9, 10]. These differences necessitate higher spatial resolution for comparable neuroanatomical detail compared to human imaging [11] but come at the cost of reduced signal‐to‐noise ratio (SNR) due to smaller voxel sizes. Traditional RF coils designed for humans are not ideal for NHPs; they must be scaled down to fit the smaller monkey head while preserving a high‐channel count for parallel imaging to minimize distortions in EPI images, which are particularly pronounced at UHF.

Several dedicated RF coil arrays have been developed for NHP brain imaging across magnetic field strengths. At 3 T, receive‐only loop arrays with 16–24 channels demonstrated substantial gains in SNR and parallel imaging performance compared with human pediatric or low‐channel‐count coils, while relying on scanner body coils or simple volume transmitters for excitation [12, 13, 14]. At 7 T, integrated transmit–receive solutions were developed, including a single‐channel large‐loop transmitter combined with 16 receive loops [15], a birdcage transmitter paired with a 16‐channel AC/DC receive array incorporating *B*_0_ shimming [16], and an 8‐channel loop transmit array combined with a high‐density 24‐channel receive array [17]. At 10.5 T, a hybrid system combining an 8‐dipole transceiver array with a high‐density 24‐channel receive array further improved SNR, deep‐brain sensitivity and parallel imaging performance [6].

While these coil systems represent significant advances for NHPs imaging at UHF, most have been designed exclusively for anesthetized subjects to facilitate baseline imaging and obtain valuable information about brain structure and function (References [18, 19, 20, 21, 22, 23], but see [24]). However, this approach limits the study of naturally occurring brain activity, behavior, and pharmacological effects. Awake fMRI, on the other hand, provides more ecologically valid conditions for studying neural processes associated with natural cognitive functions, but it requires specific mechanical configurations to maintain subject comfort [25]. This necessitates a special arrangement of coils elements that supports high‐resolution whole‐brain coverage without obstructing visual stimulus delivery or interfering with other essential experimental equipment. The combination of these factors makes developing RF coil systems for awake and task‐based NHP imaging both technically and mechanically demanding.

**FIGURE 1:**
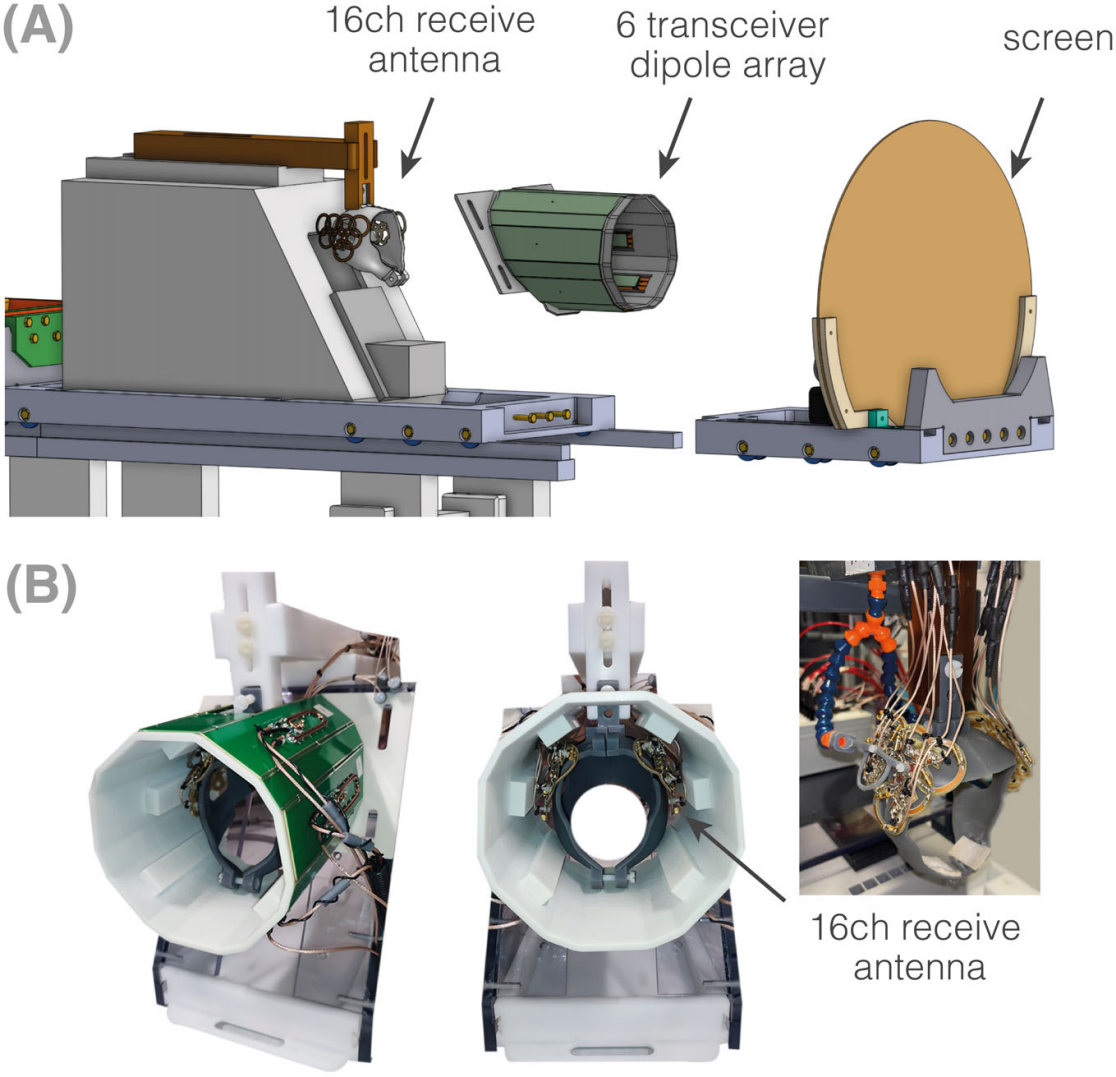
RF coil system mechanical design. (A) 3D view of the RF antenna system showing the 16‐channel receive array and 6 dipole transceivers integrated into a custom housing with visual stimulus screen. (B) Photos of the assembled device with insets showing the 16‐channel loop array.

To address these challenges, we have developed and experimentally validated a novel hybrid RF coil system specifically optimized for NHP brain imaging at 7 T (Figure 1). This system combines an outer 6‐channel preamplifier decoupled short dipole parallel‐transceive array with a 16‐loop tight‐fitting receive array.

In UHF MRI, dipole array antennas have emerged as a good solution for both transmit and receive applications [26, 27]. Studies have shown that dipoles offer greater penetration depth than loop coils [28], higher transmit efficiency, and improved uniformity of the transmit field [29]. However, traditional dipoles have limitations when used in receiving mode. They are highly sensitive to load variations and prone to strong inter‐element coupling [30], which can degrade signal quality. On the other hand, high‐density loop arrays with tightly packed elements offer improved parallel imaging capability [31, 32] and enhanced sensitivity at the periphery of the brain [1, 33]. Yet, when these loop sizes are reduced to accommodate the smaller NHP heads while maintaining a high channel count, their penetration depth is significantly limited.

To overcome these challenges, our approach incorporates transmit elements as receivers in a hybrid configuration. This design not only increases overall SNR but also combines the advantages of both dipoles and loops, with dipoles providing deeper tissue penetration and loops enhancing peripheral SNR [3, 6]. However, in such a compacted design, ensuring that the dipole and loop arrays do not interfere with each other is crucial.

In high‐density loop configurations, geometric decoupling has traditionally been employed using an optimal “soccer ball” configuration [34] where adjacent elements overlap for effective isolation. However, such overlapping is not feasible for next‐nearest‐neighbor loops. Consequently, preamplifier decoupling [35, 36, 37] has become the standard solution to reduce crosstalk in these systems. This approach uses commercial preamplifiers that transform their low input impedance into a high impedance as perceived by the receive element, effectively minimizing mutual interference between loops.

Building on this established technique, we present for the first time a dipole design which incorporates preamplifier decoupling in these dipole elements when they are in receiving mode, significantly reducing mutual coupling not only between loops but also between dipoles themselves, and most importantly, between the loops and dipoles.

In this work, we present a comprehensive description of the design, simulation, and experimental validation of our 6‐dipole and 16‐loop RF coil system. Through careful electromagnetic simulations, we optimized the dipole transceivers to achieve high $B_{1}^{+}$ homogeneity, good penetration depth, and effective preamplifier decoupling. Experimental results were compared with simulations, confirming the accuracy and effectiveness of our design. Experimental validation included bench tests and in vivo imaging, producing high‐quality anatomical images. Furthermore, functional‐MRI (fMRI) data acquisition allowed for the assessment of resting‐state networks, confirming that our system maintains reliable image quality even at submillimeter (0.75 mm isotropic) high resolutions required for detailed NHP brain studies.

## 2 Methods

### 2.1 Electromagnetic and Circuit Simulations

To design the 6‐dipole array, electromagnetic and circuit simulations helped in predicting the transmission and reception performances using the high‐frequency structural simulator (HFSS) and circuit co‐simulation from the Ansys electromagnetic suite (Ansys, PA, USA). Simulations were conducted on a 3D model of a macaque head with dielectric properties mimicking biological tissues at 300 MHz (σ = 0.78 S/m, 𝜀 = 47) [38].

A first step was to compare the transmit performances of different element types (single loop vs. multiple dipoles). $B_{1}^{+}$ maps were computed with 1 W of total power dispatched at the transmit ports, and a pseudo‐CP mode was found with phase shimming at the center of the macaque brain. After selecting the appropriate element type for effective transmission, an initial optimization of the dipole blade design was performed to maximize the $B_{1}^{+}$ field strength and penetration depth. Then, circuit co‐simulation helped in designing impedance matching circuits for fine tuning, power‐matching in transmission and noise‐matching in reception.

The simulated SNR was computed voxel‐by‐voxel as the noise covariance weighted root sum‐of‐square [34], also referred to as Roemer's SNR reconstruction [36]:

(1) $$\mathrm{SNR}=\sqrt{S_{r}^{*}\Psi^{-1}S_{r}}$$

where $S_{r}$ is the N‐column vector containing the $B_{1}^{\text{receive}}$ profiles computed from the reciprocity theorem and Ψ the noise covariance matrix.

(2) $$\begin{array}{c} B_{1}^{\text{receive}}=\frac{B_{1x}+iB_{1y}}{2} \end{array}$$

The field maps were exported from HFSS with a 2 mm isotropic resolution and postprocessing of the data was performed with a custom Python pipeline for SNR comparison with scanner measurements.

### 2.2 Mechanical Design

Coil housings were designed using Onshape (PTC, MA, USA) and manufactured with polyamide PA3200 powder filled with glass beads (Initial, Prodways Group, France) or through stereolithography (SLA) with a photopolymer resin using in‐house 3D printers (Formlabs, MA, USA). The dimensions of the dipole‐bearing cylinder were selected to fit with a sphinx position monkey chair (Figure 1A) to be compatible for both awake and anesthetized experiments. The dipole support incorporates an aperture at the top for head fixation systems such as headposts [25, 39, 40] for awake imaging or subject‐specific 3D printed masks for anesthetized experiments (Figure 1B). The RF coil was engineered to be concentrically insertable within a specific multi‐coil array $B_{0}$ shim [41]. The mechanical setup procedure consists of first positioning the NHP, followed by placement of the receive loop array, and finally sliding the transmit dipole cylinder into position, with or without the additional $B_{0}$ shim array. This coil can accommodate animal with a maximum left–right head diameter of approximately 11 cm and a maximum superior–inferior head dimension of approximately 13 cm.

### 2.3 16‐Channel Receive Loop Array

**FIGURE 2:**
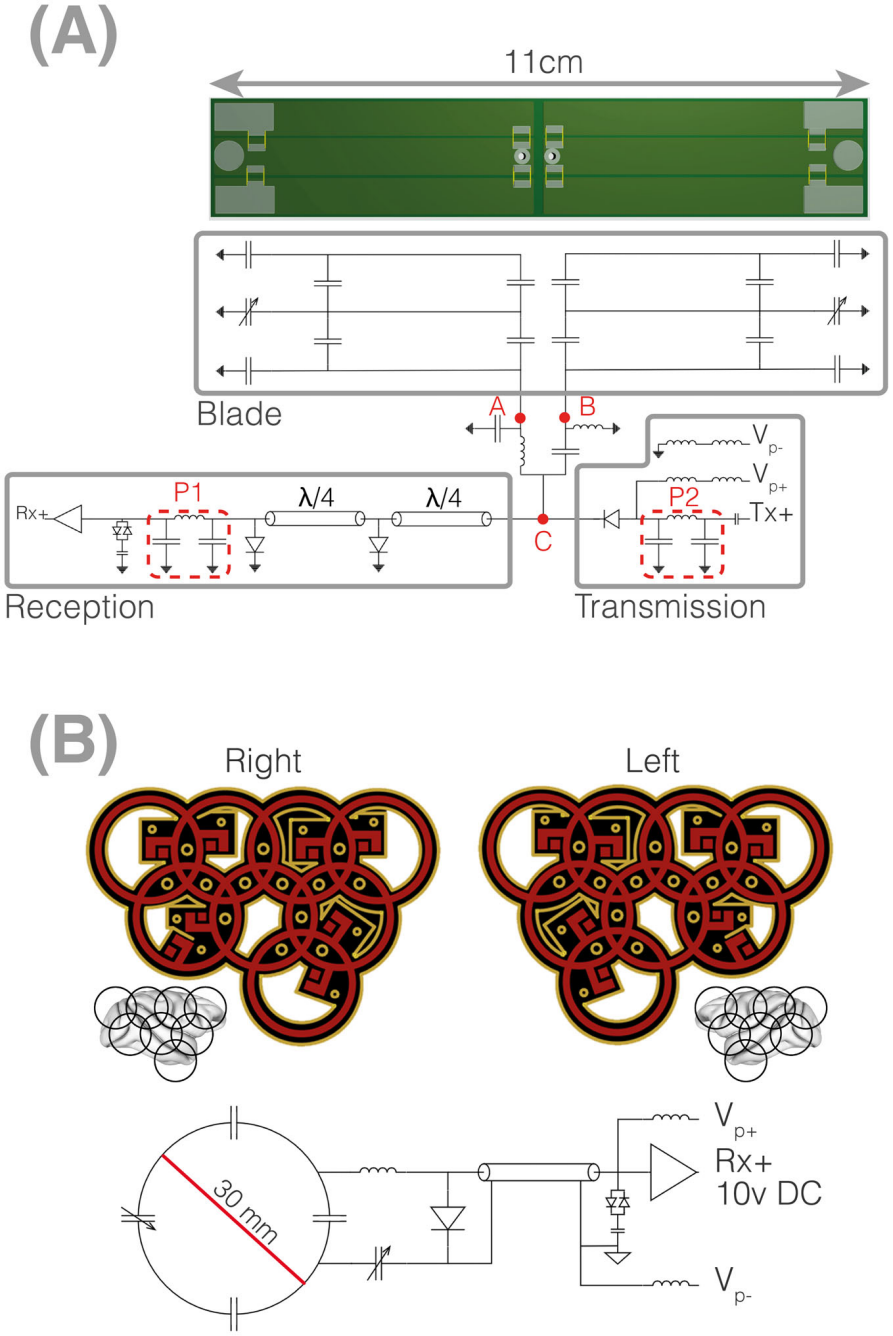
Circuit schematics of the hybrid coil system. (A) Schematic of a dipole transceiver element with matching and decoupling circuits. (B) Layout and circuit of the 16‐channel loop array.

The 16‐loop receiver array includes two eight‐loop sub‐arrays, symmetrically positioned on either side of the NHP's head. Each sub‐array contains eight loops with a diameter of 30 mm, fabricated on FR4 PCB material (35 μm copper thickness, 2 mm trace width, and 1.6 mm substrate thickness). All loops are tuned to the Larmor frequency at 297.2 MHz. Geometric decoupling between adjacent loops is achieved using adjustable copper wire bridges placed at the overlap region, which are manually adjusted during bench measurements to iteratively minimize S_12_ mutual coupling. Coupling between more distant loops is minimized through preamplifier decoupling circuits. Active detuning is accomplished using pin diode circuits to detune the loops during the transmission phase (Figure 2B). To mitigate electromagnetic interference and save space, the low input impedance preamplifiers (WM7MRP, WanTcom, Chanhassen, MN) are located outside the coil, mounted on top of the chair, with cables routed through the upper opening of the dipole cylinder, ensuring they are inaccessible to an awake NHP and remain outside the visual field. Cable traps were installed between the loops and their preamplifiers on *λ*‐long coaxial cables to prevent sheath currents induced during transmission by the dipoles (*λ* ≈ 65 cm at 300 MHz, associated to an estimated insertion loss of 0.70 dB). Two cable traps were placed at *λ*/4 and *λ*/2 from each receiving loop inside the dipole array. Each trap has a variable capacitor, tuned to minimize the S12 measurement at both ends of the cable trap with common ground. The preamplifiers have a 1.5 Ω input impedance, 0.45 dB noise figure and 28 dB gain, and are DC‐powered with 10 V across the RF coaxial cables.

### 2.4 6‐Dipole Transceiver Array

A 17‐cm diameter and 22‐cm long beveled cylinder contains 6 dipoles around the NHP head, with two lower dipoles shifted in the scanner bore axis direction (Figure 1). The dipole blades are accessible to allow adjustment of variable capacitors to fine‐tune the coil. Preamplifiers, matching circuits, and T/R switches are located on the outer side of each dipole PCB, with cables running from the front to connect to ODU plugs (ODU, Mühldorf, Germany) for transmission and reception.

The dipoles were fabricated on standard FR4 PCB by a manufacturer (Safe PCB, France). A 3‐split 11‐cm long and 10‐mm elevated blade design (Figure 2A) was chosen for its simulated $B_{1}^{+}$ field strength and reasonable impedance stability across varying loading conditions. The 6 dipoles were tuned with four 4.6 pF capacitors and two variable capacitors (range 0.8–8 pF) (Knowles‐Syfer, Norwich, UK). A lumped‐element balun, tuned to 297.2 MHz, is used as an impedance transformation circuit for the blade (A and B point in Figure 2A) and acts as a current reduction element in the blade during reception when the impedance presented at point C by the quarter‐wavelength line is low. This configuration achieves a high blocking impedance as seen by the receiving element. Consequently, the six dipoles are decoupled in reception via preamplifier‐decoupling, with no specific inter‐dipole decoupling implemented during transmission apart from cable traps to suppress sheath current along the cables. Impedance transformation circuits (P1 and P2 in Figure 2A) are employed separately for transmission and reception, allowing to respectively power‐match and noise‐match the impedance to 50 Ω. T/R switches consist in two *λ*/4 lines and 3 PIN diodes (MA4P1250NM‐1072T, MACOM, Lowell, MA, USA). In transmission mode, a subject‐optimized phase shimming aligns the 6 TX phases at the center of the NHP brain to achieve good $B_{1}^{+}$ efficiency.

### 2.5 Bench Measurements

Coil tuning, detuning, and coupling were evaluated on the bench using a 4‐port VNA (Rohde & Schwarz, Germany). Measurements were done with a single‐loop probe or with a decoupled B‐field double probe. Fine tuning measurements were conducted with the entire setup and a NHP brain phantom with a conductivity σ = 0.78 S/m and permittivity 𝜀 = 47. Spurious preamplifier oscillations and Quality (Q) factors [42] were examined in a RF shielded box (1720, JRE test, USA) with a VNA, signal generator (Rohde & Schwarz, Germany), and signal and spectrum analyzer (Rohde & Schwarz, Germany).

### 2.6 Animal Experiments

Two adult monkeys ( *Macaca mulatta* , 1 male and 1 female, 9–10 kg) were examined at 7 T in the sphinx position, with their head maintained with a subject‐specific 3D printed helmet. All experiments were conducted in strict accordance with European (EC/2010/63) and French regulations (French Act Rural Code R214‐87 to 126) and were approved by the Ethics committee and the French ministry of higher education and research.

Animals were prepared for imaging using comprehensive protocols ensuring their welfare throughout the procedure. They were fasted for 12–15 h prior to sedation. The initial sedation was induced with a mixture of Ketamine (3 mg/kg) and Dexdomitor (0.015 mg/kg) and was maintained under anesthesia with intravenous slow infusion of Alfaxan (0.10 mg/kg/min). Animals were intubated and ventilated during the entire experiment using a ventilation machine (Aestiva/5 MRI, GE Healthcare, USA). A flexible heating pad maintained the animal's temperature at 38°C. Heart rate, respiratory rates, blood pressure, blood oxygen saturation, etCO2, and body temperature were monitored and recorded throughout the entire imaging session using an MRI compatible monitor (IRADIMED, USA).

### 2.7 Acquisition Protocol

Experiments were performed using a 7 T MRI scanner (MAGNETOM, Siemens, Erlangen, Germany) equipped with an 8‐channel parallel transmission system with 1 kW peak power per channel, and a “SC72” body gradient set operating at a slew rate of 200 mT/m/s and 70 mT/m maximal strength.

The calculation of the 6 complex transmit B1 fields was based on a multi‐slice interferometric XFL acquisition [43, 44, 45] (3 mm isotropic resolution, FOV = 64 × 64 × 32 mm^3^, TE = 2 ms, TR = 10 s); the resulting B1‐maps were used for phase shimming with a custom MATLAB code at the beginning of each imaging session.

To compute SNR, a 1.5 mm isotropic 3D gradient recalled echo (GRE) sequence was acquired (TR/TE = 15/2 ms, FA = 7°, FOV = 128 × 128 × 64 mm^3^). Noise measurements were acquired using the same GRE sequence with 0 V excitation, yielding the noise covariance matrix.

$B_{0}$ field maps were acquired with a 1.5 mm‐isotropic 3‐echo GRE sequence (TR = 15 ms, TEs = 1.8, 6.2, 8.5 ms, FA = 7°).

Anatomical images were acquired using a T2‐weigthed Turbo Spin Echo (TSE) sequence (0.4 × 0.4 × 1.2 mm^3^, FOV = 152 × 152 mm^2^ (384 × 384), TR/TE = 6620/80 ms, iPAT = 3, FA = 60°, BW = 121 Hz/px) and a T1‐weighted MP2RAGE sequence (0.75 mm iso, FOV = 115 × 168 mm^2^ (154 × 224), TR/TE = 6000/3.05 ms, iPAT = 3, FA = 4°, BW = 240 Hz/px).

We acquired resting‐state fMRI images with a CMRR 2D SMS/Multi‐Band EPI sequence [46] at a 1 mm isotropic resolution (TR/TE = 1960/24 ms, iPAT × MB = 3 × 2, PF = 7/8, BW = 1158 Hz/px) and a 0.75 mm isotropic resolution (TR/TE = 1460/19.6 ms, iPAT × MB = 3 × 2, PF = 6/8, BW = 1184 Hz/px). Data were acquired in a single run per subject, lasting 1643 s and comprising 1125 functional volumes.

### 2.8 SNR, tSNR, g‐Factor, and $B_{1}^{+}$ Maps: Experimental Evaluation

The GRE data were postprocessed via a custom‐written MATLAB code, and SNR was reconstructed following Kellman's [47] method (without low SNR correction, as the measured SNR far exceeded 100), as a weighted sum‐of‐square‐combination of the image data, considering the noise covariance matrix [36]. Data were then corrected for transmit inhomogeneities using:

(3) $$\begin{array}{c} \mathrm{SN}R_{\text{FAcor}}=\mathrm{SNR}\frac{1-E_{1}\cos\left(\mathrm{FA}\right)}{\left(1-E_{1}\right)\sin\left(\mathrm{FA}\right)} \end{array}$$

with $E_{1}=e^{-\frac{\mathrm{TR}}{T1}}$ and FA the flip angle.

Temporal signal‐to‐noise ratio (tSNR) was calculated from non‐preprocessed images by dividing the mean signal intensity of each voxel by its temporal standard deviation, measured across 600 time points.

The g‐factor maps were calculated from the SENSE [32] equations for one‐dimensional and 2D k‐space undersampling, using individual SNR maps as sensitivity profiles and noise covariance matrix. The data for the g‐factor maps were obtained with a fully sampled 1.5‐mm isotropic 3D‐GRE sequence. The SNR of the accelerated images ($\mathrm{SN}R_{\mathrm{acc}}$) was calculated from the SNR without acceleration ($\mathrm{SN}R_{\text{unacc}}$) as follows:

(4) $$\begin{array}{c} \mathrm{SN}R_{\mathrm{acc}}=\frac{\mathrm{SN}R_{\text{unacc}}}{g\sqrt{R}} \end{array}$$

where *R* is the reduction (acceleration) factor for k‐space undersampling [32]. For g‐factor computation, the FOV (120 × 120 × 68) was tightly fit to the head, which maximizes aliasing in the accelerated image. The resulting maps were then brain masked for visualization purposes.

The complex individual $B_{1}^{+}$ maps obtained after phase shimming were used for $B_{1}^{+}$ calculation and normalized to the RF power level at the coil input to obtain $B_{1}^{+}$ maps for 1 W of total injected power into the system. The homogeneity of the $B_{1}^{+}$ field maps was assessed by calculating the relative standard deviation (RSD) (i.e., the ratio of the standard deviation to the mean) within the volume of interest.

### 2.9 Functional Data Processing

Functional images preprocessing was performed using a custom pipeline relying on FSL [48], ANTs [49], AFNI [50] and FreeSurfer [51] using nipype [52]. Seed‐based correlation analysis was performed with Nilearn [53].

Images were first denoised using NORDIC [54], despiked, motion corrected, and slice time corrected using AFNI, and EPI distortion corrected using FSL FUGUE. After bias field correction, anatomical and functional images were nonlinearly warped into the MEBRAINS [55] atlas using ANTs and the JIP analysis toolkit. Images were spatially smoothed (FWHM = 2 mm), linear detrended and denoised using a linear regression approach [56, 57] including motion regressors, as well as a 5‐component nuisance regressor of the masked white matter and cerebrospinal fluid regions [58]. Band‐pass filtering (0.008–0.1 Hz) [59] was used to remove low‐frequency drifts and high‐frequency noise components, following previously established procedures [60, 61].

Posterior cingulate/precuneus cortex (pC/PCC) seeds were anatomically defined according to the CHARM [62] atlas. Voxel‐wise correlation maps were generated by computing Pearson correlations between the averaged PCC time series and all brain voxels. Fisher‐z transformed correlations were thresholded with the null hypothesis of no correlation at *p* < 10^−5^ and were projected from volume to the MEBRAINS cortical surface [55].

## 3 Results

### 3.1 Transmit Performances

**FIGURE 3:**
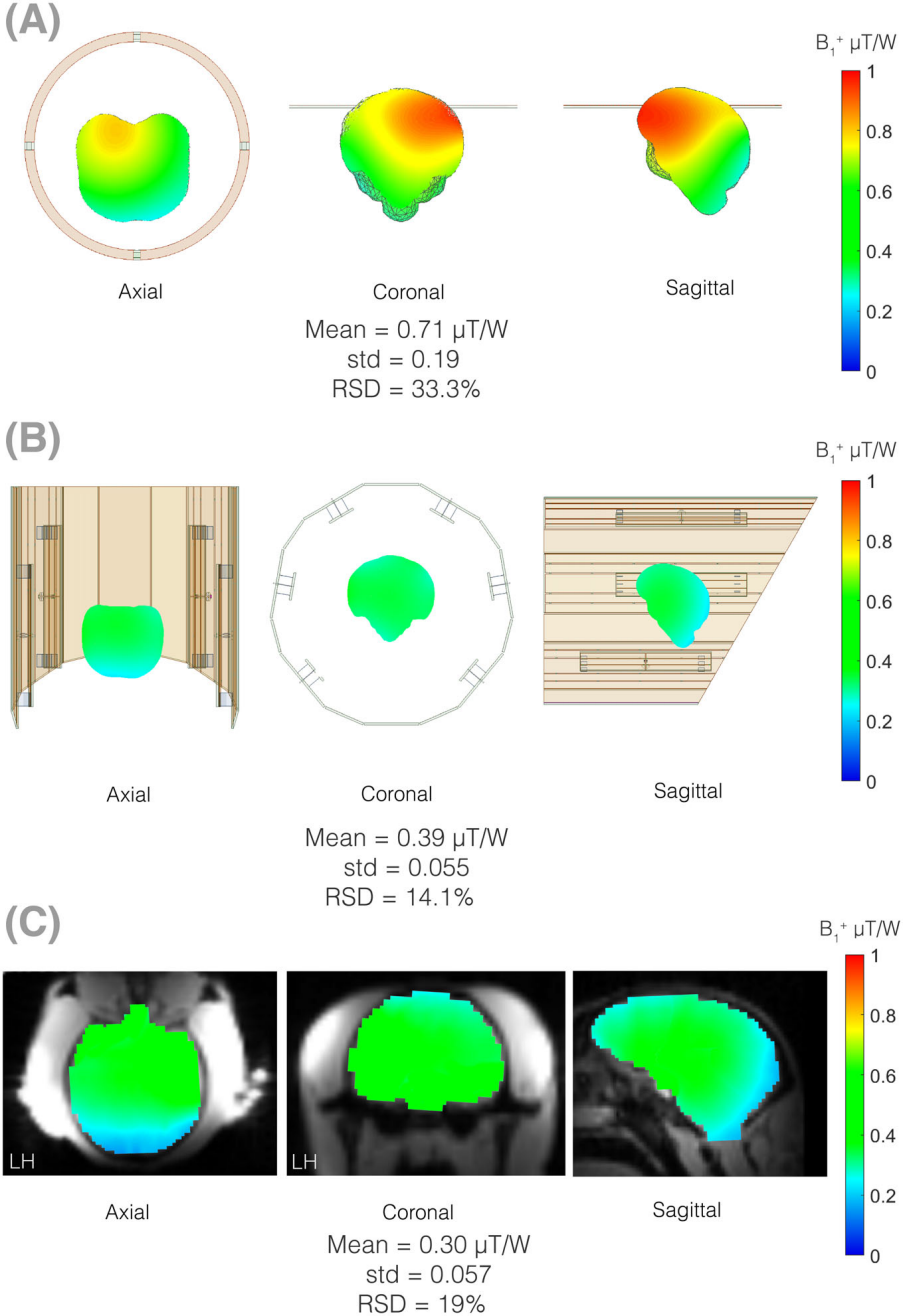
Transmit performance: Simulation versus experiment. (A) Simulated $B_{1}^{+}$ map from an 11‐cm single loop at 1 W excitation power. (B) Simulated $B_{1}^{+}$ map from the 6‐dipole array at 1 W total system power. (C) Experimental $B_{1}^{+}$ map acquired in vivo with a subject‐specific phase shimming in pseudo‐CP mode.

We first compared $B_{1}^{+}$ performances of an 11‐cm loop over a 6‐dipole array. Specifically, the simulated $B_{1}^{+}$ map for the single loop exhibits pronounced signal inhomogeneity (Figure 3A). In contrast, the 6‐dipole array provided a more uniform $B_{1}^{+}$ distribution throughout the brain, characterized by reduced variability with narrower $B_{1}^{+}$ value distribution and almost two times lower quartile coefficient of dispersion (QCD), 0.23 for the loop against 0.12 for the dipoles. The RSD is more than two times higher using the single loop than the dipole array (33.3% against 14.1%) (Figure 3B). However, the mean $B_{1}^{+}$ over the brain is higher with the loop than with the dipole array (0.71 μT/√W against 0.39 μT/√W). The lower 6‐dipole transmit efficiency is not critical as our RF power amplifiers provide sufficient peak power to reach whatever flip angle is needed in the whole brain within reasonable pulse time duration.

Experimental $B_{1}^{+}$ mapping of the dipole array closely matched simulation results, with the measured experimental $B_{1}^{+}$ distribution demonstrating a relatively uniform coverage within the brain region (mean experimental $B_{1}^{+}$: 0.30 μT/√W, RSD = 19%) (Figure 3C). Subject‐specific $B_{1}^{+}$ phase shimming was used both experimentally and in simulation, providing substantial brain coverage. However, the posterior part of the brain lacks excitation, possibly due to head position along the z‐axis which could be tackled with a repositioning or using dynamic pTx (Figure S2) [63].

### 3.2 Reception Performances

The six channels of the dipole transceiver were tuned and matched such that the return loss is inferior to −10 dB in both transmit and receive mode. Nearest neighbor coupling in transmit mode averages −12.3 dB. The 30 mm receive loops were tuned and matched to approximately −18 dB with average coupling to non‐overlapped nearest neighbor of −14 dB; their decoupling is largely impacted by cable routing and cable traps tuning and position. Measured Q‐ratio is 2.94. Active loop decoupling performance, measured with a double probe, reaches on average −37 dB of isolation.

**FIGURE 4:**
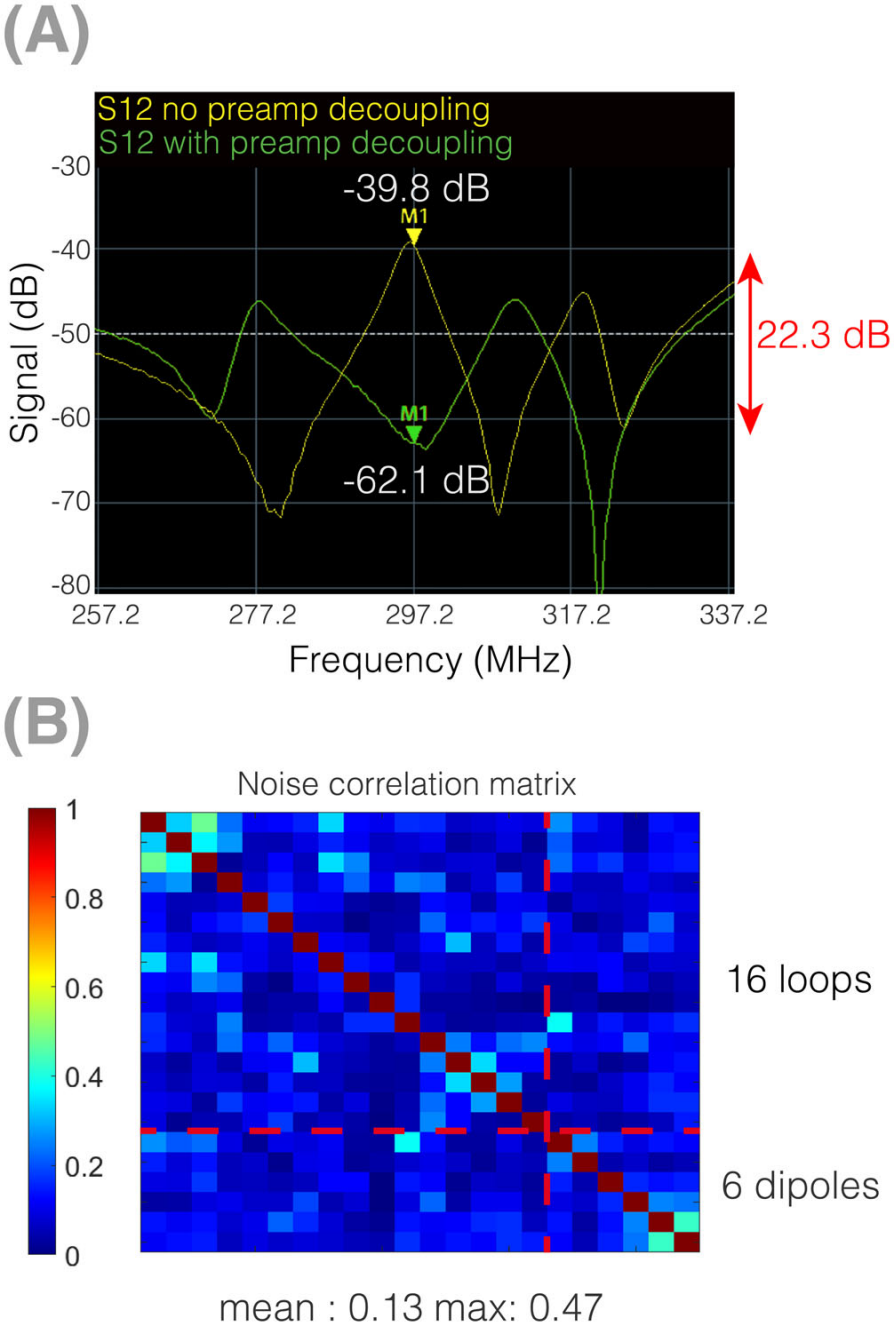
Validation of preamplifier decoupling efficacy. (A) S12 measurements using a double probe near a dipole element with and without preamplifier decoupling showing a difference of 22.3 dB. (B) Noise correlation matrix across all 22 channels (16 loops + 6 dipoles), showing a mean value of 0.13.

S12 measurements using a double probe near a dipole element show a significant difference of 22.3 dB with and without preamplifier decoupling (Figure 4A). Complete bench characterization of both the loop and dipole elements is reported in Tables S1 and S2. The efficiency of preamplifier decoupling is evaluated experimentally with a noise correlation matrix, indicating a mean correlation of 0.13 and a maximum correlation of 0.47 (Figure 4B). Repeated computation of noise correlation matrices demonstrates that inter‐element coupling remains low and stable across different sessions (Figure S1).

**FIGURE 5:**
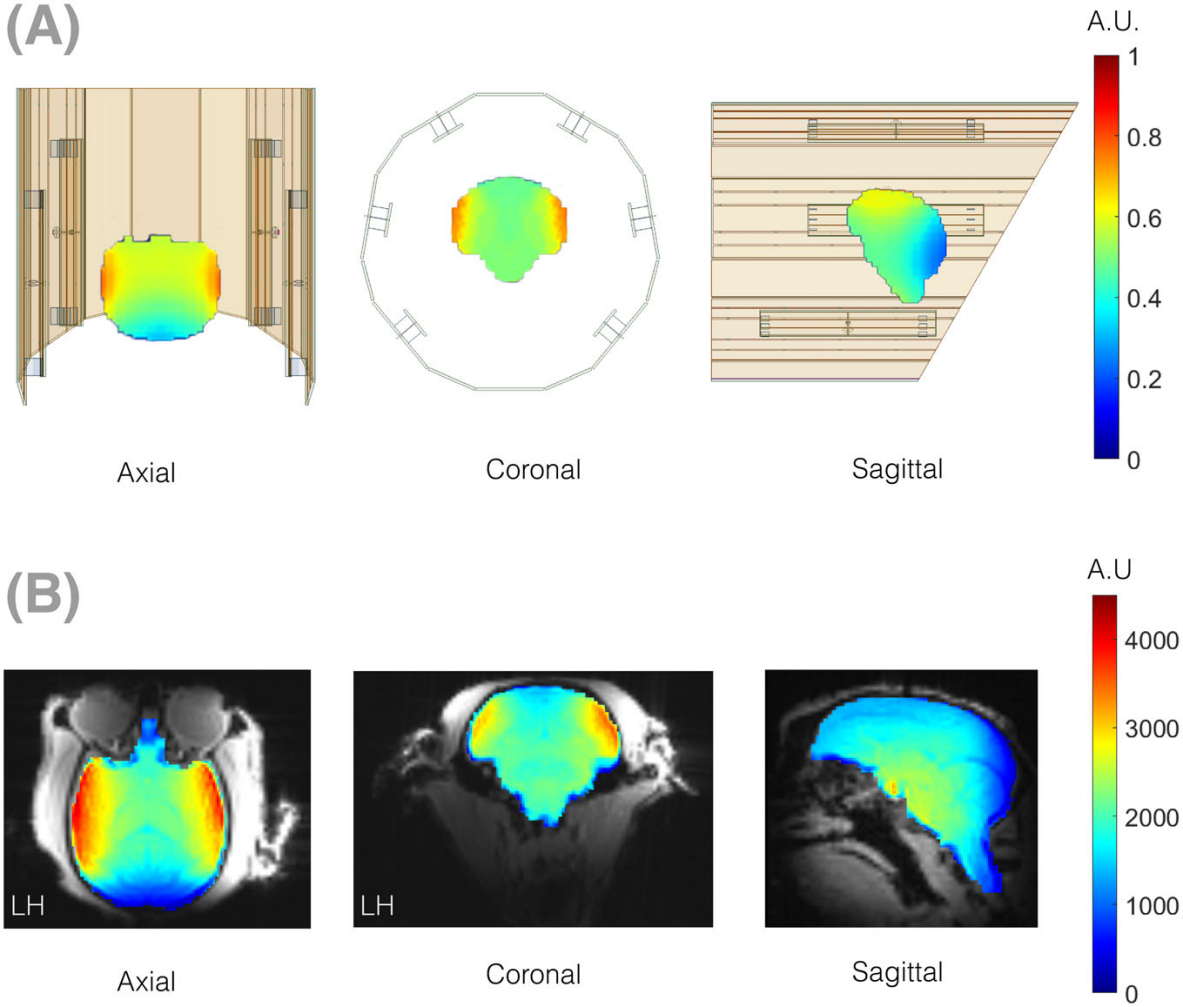
Dipole array reception performance: Simulation versus experiment. (A) Simulated SNR map from the 6‐dipole array. (B) Experimental SNR maps from the 6‐dipole array.

Simulated SNR for the 6‐dipole array indicates high SNR values at the brain's periphery but demonstrates good penetration depth (Figure 5A). In vivo experiments confirmed the simulated SNR profile predictions with good coverage (Figure 5B).

**FIGURE 6:**
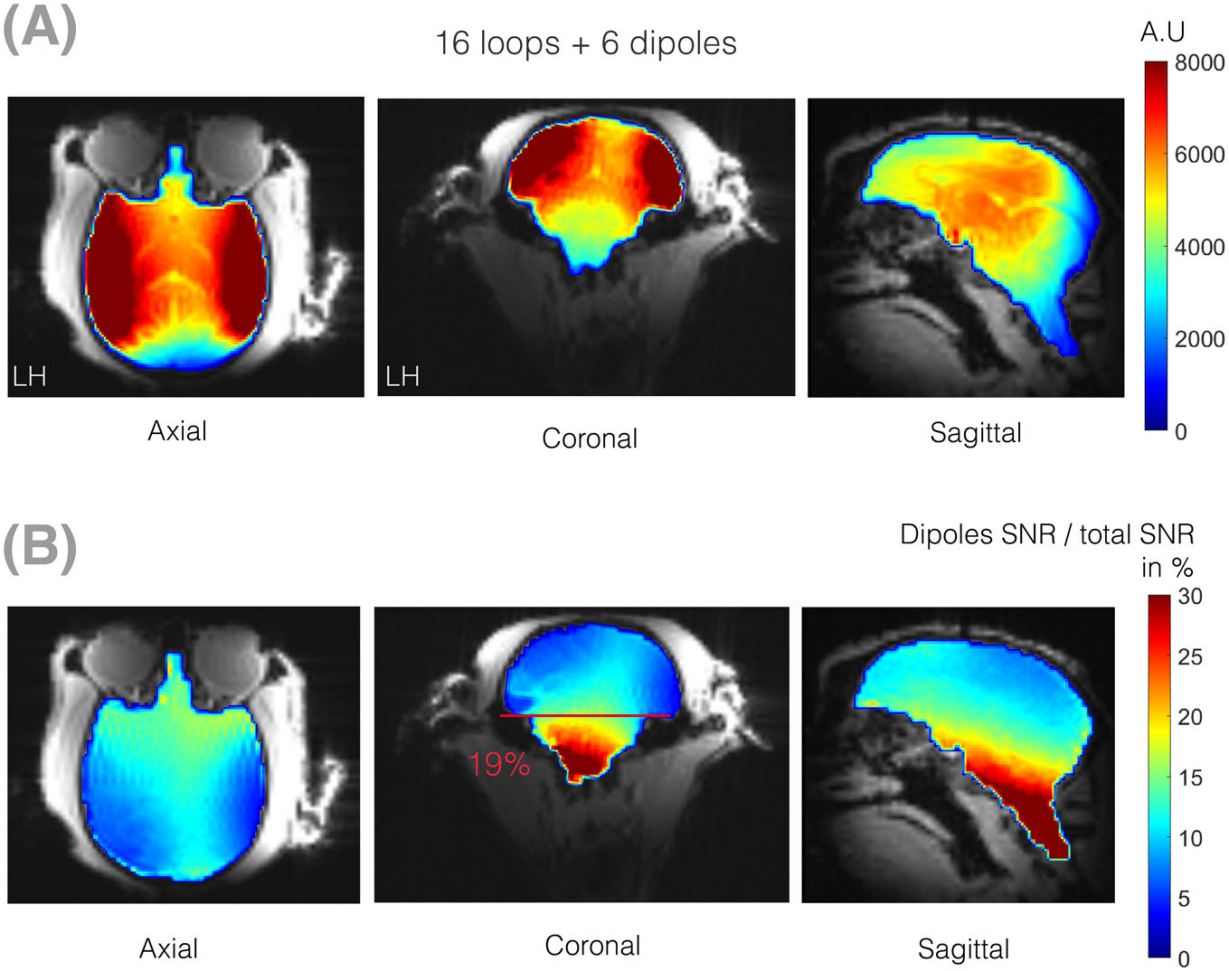
Hybrid system SNR and dipole contribution. (A) Overall SNR maps from the entire RF system. (B) Contribution of dipoles to total SNR, showing 19% contribution of SNR to the ventral part of the brain (below the red line).

SNR mapping with the complete system indicates high peripheral SNR, high global SNR, and good coverage of the brain (Figure 6A). As anticipated, SNR decreases rapidly as the distance from the loop arrays increases, with greatest SNR in the scalp and muscle regions. However, the 6‐dipole array significantly enhances the central and ventral SNR, with an average of 19% contribution over the ventral part of the brain (Figure 6B).

**FIGURE 7:**
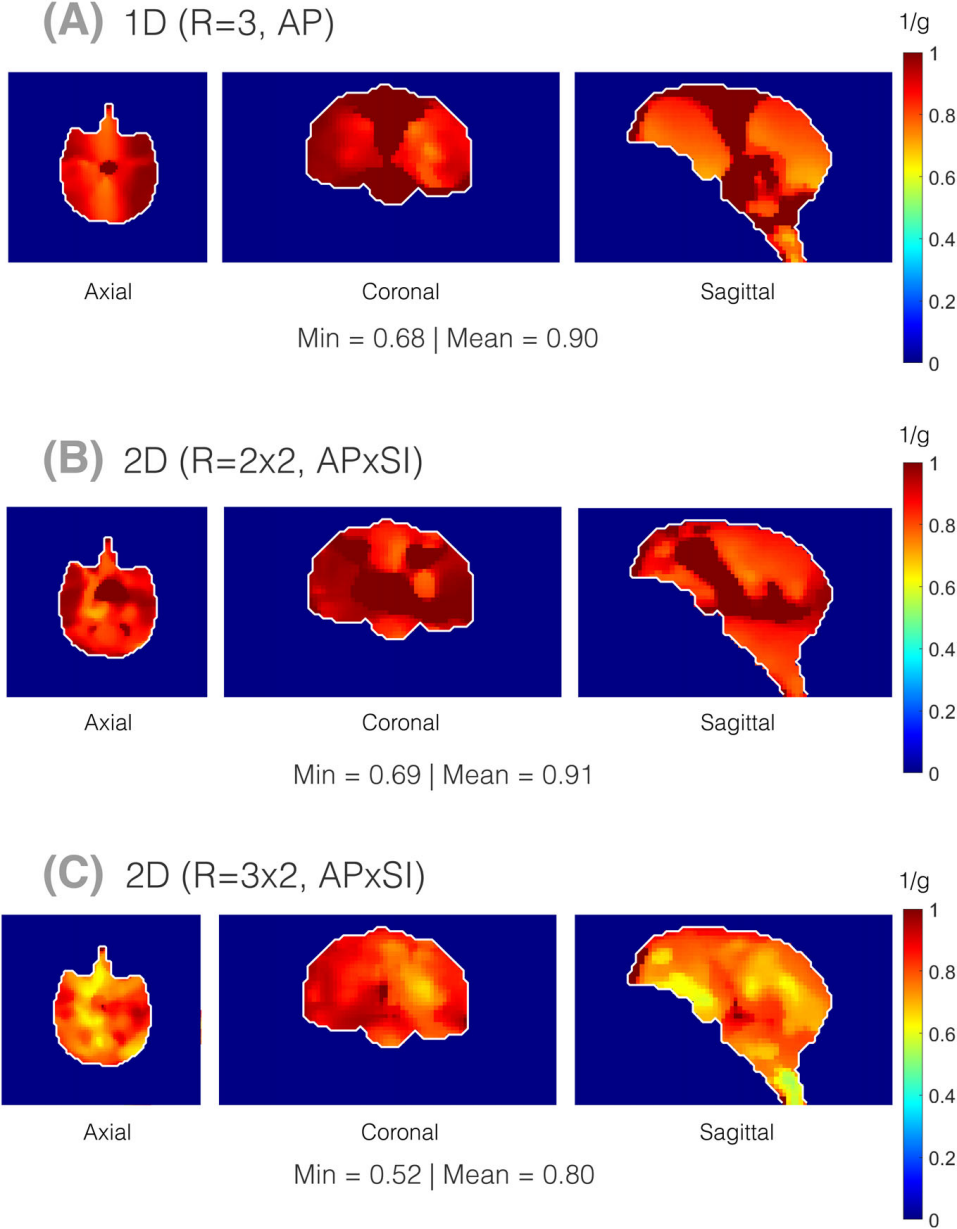
Parallel imaging acceleration performance. Inverse g‐factor maps for: (A) 1D acceleration of 3 in the AP direction; (B) 2D acceleration of 2 in the AP direction and 2 in the SI direction; (C) 2D acceleration of 3 in the AP and 2 in the SI directions. A, anterior; P, posterior; S, superior; I, inferior.

Computed 1/g‐factor maps demonstrate that the coil provides robust acceleration capabilities for 1D acceleration of 3 in the AP direction (Figure 7A) with mean 1/g of 0.90. Two‐dimensional accelerations *R* = 2 × 2 (AP × SI) and *R* = 3 × 2 (AP × SI) also shows good acceleration performances with a mean 1/g of 0.91 and 0.80 respectively (Figure 7B,C). The anterior–posterior (AP) direction is where more loops are present, making it the axis with the highest potential for acceleration. On the other hand, the left–right (LR) axis is less optimal for acceleration due to the design of the coil.

### 3.3 Image Quality

**FIGURE 8:**
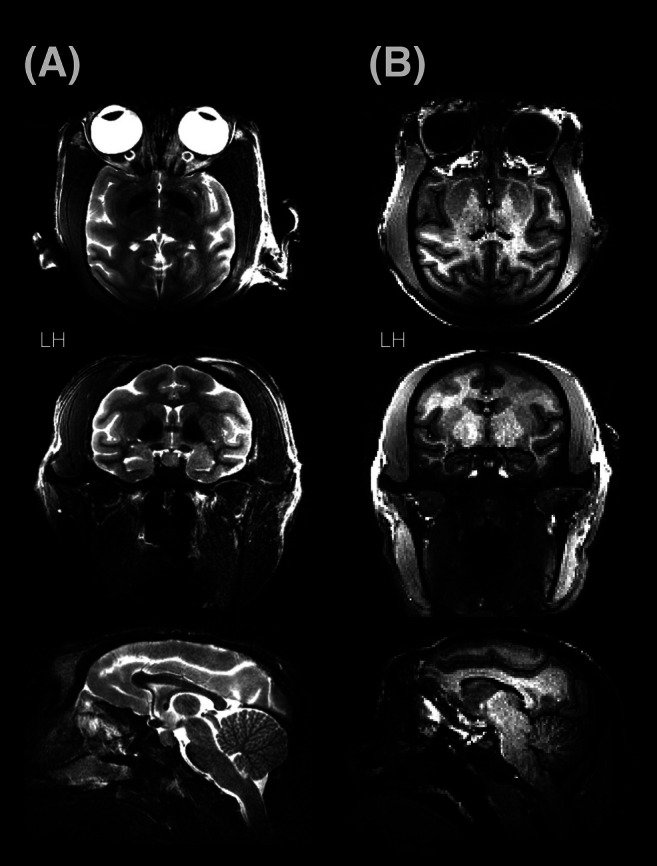
High‐resolution anatomical image quality from the hybrid coil. (A) Raw T2‐w TSE image (0.4 × 0.4 × 1.2 mm^3^). (B) Raw T1‐w MP2RAGE image (0.75 mm isotropic).

High‐resolution anatomical images acquired using T2‐weighted TSE and T1‐weighted MP2RAGE sequences displayed good structural details and clear delineation of anatomical structures (Figure 8). Adequate whole brain coverage is achieved; however, the lack of excitation in the posterior region of the brain is observed in both images, particularly the MP2RAGE.

**FIGURE 9:**
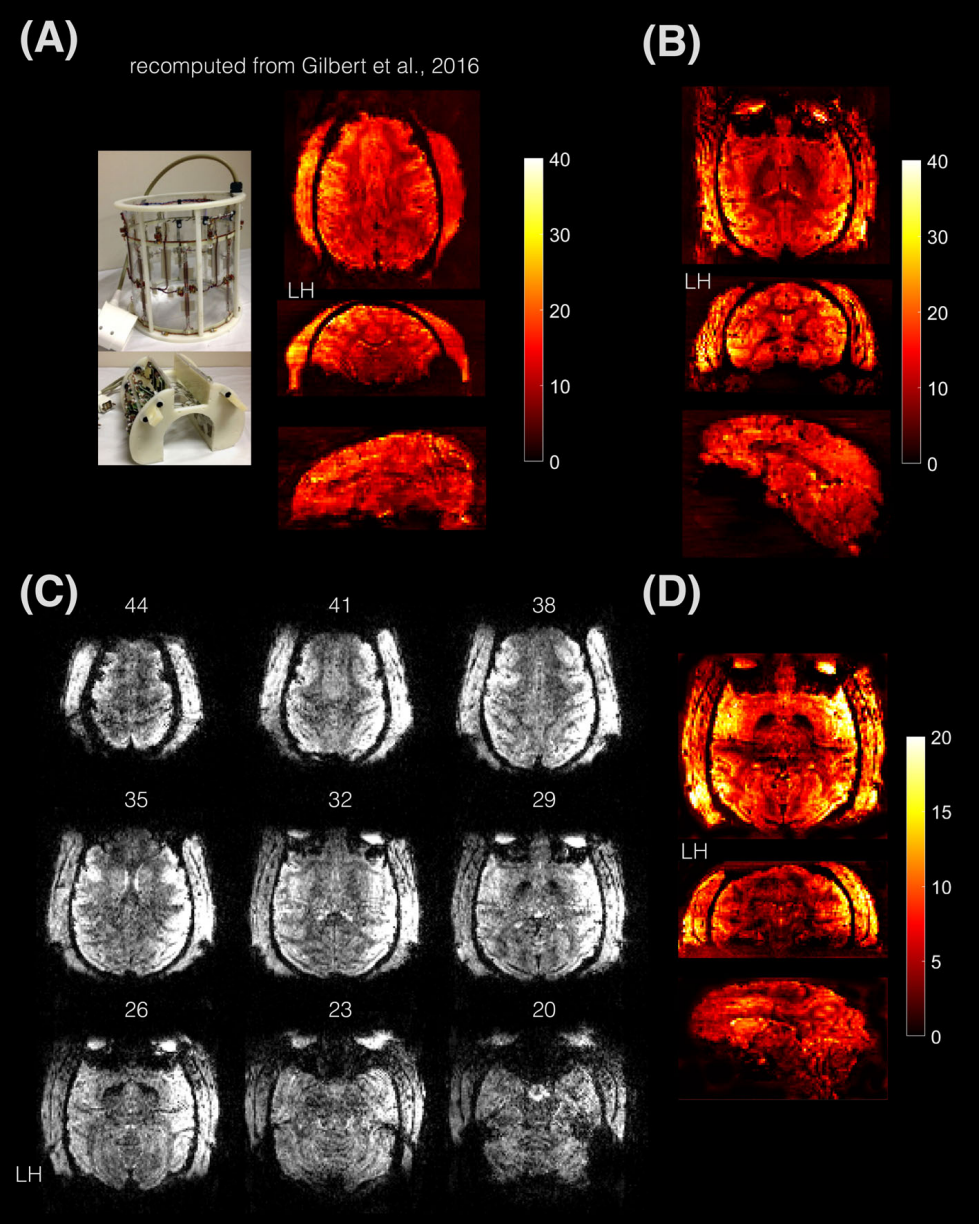
tSNR comparison at 1 mm resolution and functional MRI image quality from the hybrid coil at submillimeter resolution. (A) tSNR map computed from 600 TR acquisition (TR/TE = 1000/18 ms, *R* = 2 × 2, PF = 3/4, BW = 1680 Hz/px) at 1 mm isotropic resolution adapted from Gilbert et al. [17] accessible on the PRIME‐DE database. (B) tSNR map derived from our 600 TR EPI acquisition (TR/TE = 1960/24 ms, *R* = 2 × 3) at 1 mm isotropic resolution from the hybrid coil. (C) Raw EPI images from the 2D‐SMS acquisition at 0.75 mm isotropic resolution from the hybrid coil. Slice numbers are indicated on top of each slice. (D) tSNR map derived from 600 TR acquisition at 0.75 mm isotropic resolution across the three planes.

To evaluate functional image quality of our system, we compared our tSNR map derived from 600 TR EPI raw images at a 1 mm isotropic resolution (Figure 9B) with a tSNR map recomputed from the publicly available data in the PRIME‐DE database corresponding to a published 8 Tx/24 Rx coil [17] (Figure 9A). Although our system uses only 22 receive elements, with a higher acceleration factor (2 × 3 vs. 2 × 2) and longer TE (24 vs. 18 ms), the resulting tSNR is comparable to that of the reference coil.

High‐resolution SMS‐EPI (0.75 mm isotropic) images demonstrated good image quality, with clear anatomical definition and minimal distortions, even before undergoing any denoising or preprocessing steps (Figure 9C). tSNR mapping, obtained from a 600 TR acquisition, further indicated high‐quality image data with high tSNR across the brain for high resolution fMRI (Figure 9D).

### 3.4 Resting State fMRI

**FIGURE 10:**
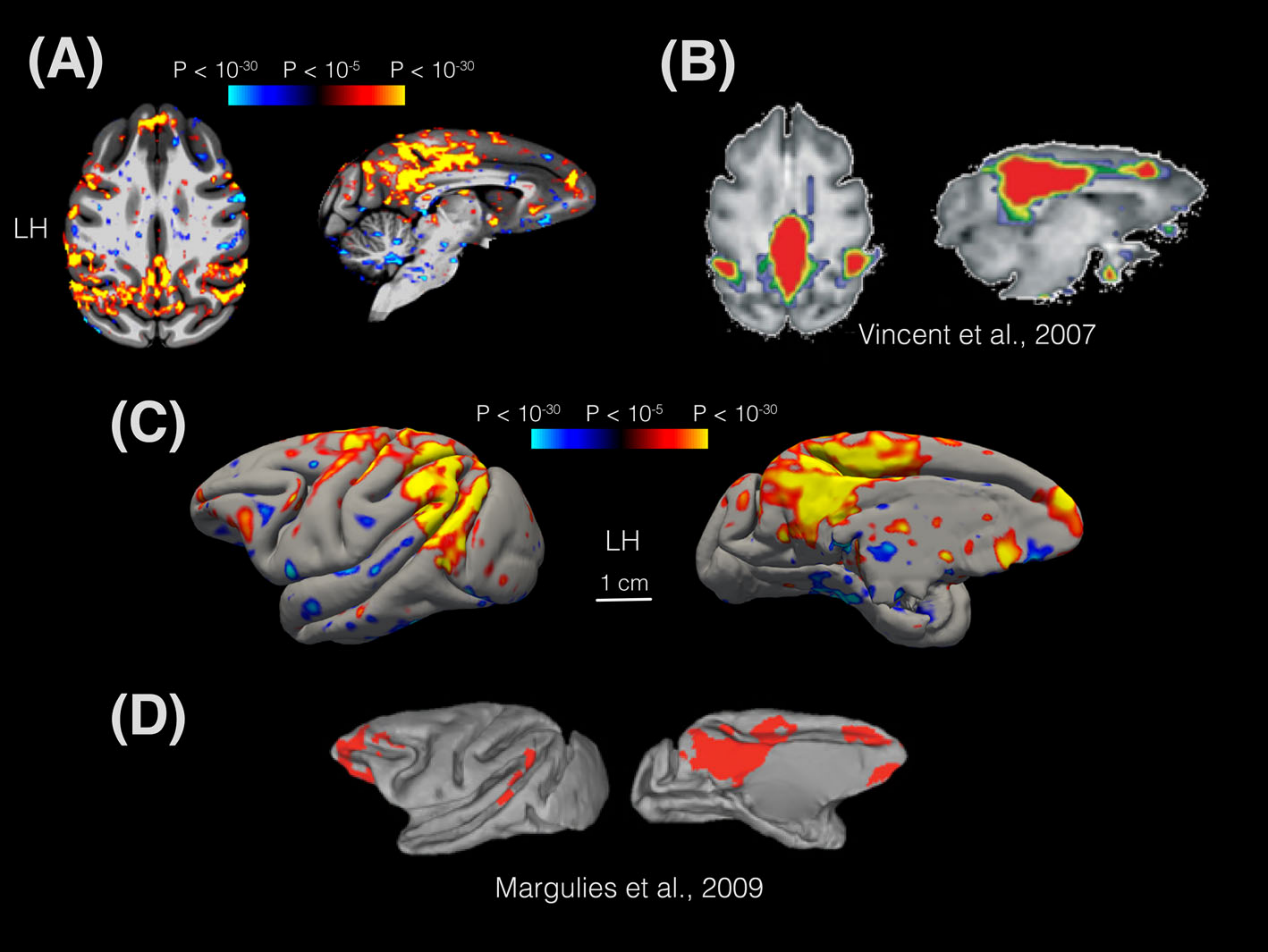
Default mode network detected by our hybrid coil at submillimeter resolution matches established primate research. (A, C) Volumetric and surface‐projected connectivity maps from the PCC seed. (B, D) Corresponding maps from the literature: Volumetric map adapted from Vincent et al. [64] (B), surface‐projected map adapted from Margulies et al. [65] (D).

Resting‐state functional connectivity analyses using a posterior cingulate cortex (PCC) seed revealed bilateral correlation patterns across the macaque cortex. Notable connectivity was observed in regions including the dorsomedial prefrontal cortex, ventromedial prefrontal cortex, and lateral temporoparietal cortex. The topography of these networks closely matches the expected default mode network architecture, with peak connectivity centered in medial and lateral associative regions, as seen in both axial and sagittal views (Figure 10A,C). These spatial patterns are highly consistent with previous macaque fMRI studies (Figure 10B,D) [64, 65, 66] and further confirm the system's capability for high quality imaging and to accurately capture functional networks.

## 4 Discussion

Our custom‐designed, tailored hybrid RF coil system for NHP brain imaging at 7 T successfully addresses several critical challenges associated with ultra‐high‐field MRI of NHPs. This system demonstrated excellent performance across multiple metrics, including high $B_{1}^{+}$ homogeneity, good SNR distribution, and parallel imaging performances. The dipole technology features short transceiver dipoles, preamplifier‐decoupled in reception mode. By combining a 6‐channel transceive dipole array with a 16‐channel loop receive array in an integrated design, we have created a versatile platform capable of high‐resolution structural and functional neuroimaging in the macaque.

In terms of transmit performances, the simulated and experimental results demonstrated that our dipole array provided substantially more uniform $B_{1}^{+}$ field than the single loop configuration, which is consistent with previous literature emphasizing the advantages of dipoles for deeper penetration and more homogeneous field at 7 T and beyond [28]. While the mean $B_{1}^{+}$ value over the brain volume was somewhat lower with dipoles compared to the single loop, the significantly reduced RSD translates directly to more consistent excitation across the entire brain volume. This is particularly valuable for UHF applications where standing wave behavior can otherwise produce severe excitation variations.

The design constraints influencing our transmit array selection were multifaceted. A traditional transmit loop could potentially obstruct the visual field, hindering functional imaging experiments requiring visual stimulus delivery. Additionally, our coil needed to be compatible with a specific $B_{0}$ shim multi‐coil array used in our 7 T system, the external diameter of the dipoles array was fixed at 18 cm, and the transmit array needed to be shielded to prevent interactions between the coil and the shim set. The proximity of this shielding necessitated avoiding the use of a transmit loop array, as such a ground plane would significantly decrease the performance of the transmit loop.

For receive performances, our implementation of preamplifier decoupled dipoles in the reception mode represents a key strength. The S12 measurements showed up to 22.3 dB improvement with preamplifier decoupling, and noise correlation analysis showed a low mean noise correlation of 0.13 between all receiving channels, which is comparable to previous multi‐channel coils designed for NHP imaging (0.22 [13] or 0.12 [17] for previous multi‐channel coils, 22 channels at 3 T and 24 channels at 7 T, respectively).

The complementary sensitivity contributions from dipoles and loops in our hybrid design create a favorable SNR distribution throughout the brain volume. While the 16‐channel loop array offered strong peripheral SNR, the contribution of more than 19% from the dipoles to ventral and deeper brain SNR underscores the benefit of hybrid arrays. A comparable hybrid antenna at 10.5 T shows a 24% contribution from the dipoles in a deep brain region [6]. These findings are comparable with other hybrid setups used at 7 T [29, 67]. Especially, our hybrid coil achieves comparable tSNR [17] despite using fewer receive elements, a higher acceleration factor, and a longer TE.

Due to manufacturing and space limitations, we have opted to maintain the number of loops at 16. Despite this, our hybrid coil demonstrated robust parallel imaging performance across multiple acceleration scenarios, enabled up to 2 × 3 (Multiband × GRAPPA) accelerations for 0.75 mm isotropic resting‐state fMRI with a short TR of 1.46 s for whole‐brain submillimeter fMRI in NHPs. This spatial resolution is higher than the typical 1–1.5 mm isotropic resolutions used in conventional 7 T NHP studies [17, 68]. While an SNR reduction relative to those coarser protocols is expected at this fine voxel size, we were able to detect the canonical default‐mode network, demonstrating the feasibility of submillimeter functional imaging with this coil. Future work may explore whether increasing the number of receive channels could further improve performance [13].

To detect the default mode network from the 0.75 mm isotropic fMRI data, we used the PCC as the seed region. The observed correlation patterns included the dorsal medial prefrontal cortex (dmPFC), the ventromedial prefrontal cortex, and the lateral temporoparietal regions. This pattern is broadly consistent with the macaque homolog of the default mode network. While previous studies have reported variable presence of the dmPFC node in the macaque default mode network, with some identifying strong functional connectivity [64, 65, 66] when seeded from the PCC and others reporting weak [69] or even absent connectivity [70], our data consistently revealed a clear and strong functional coupling between the PCC and dmPFC in anesthetized subjects. This enhanced sensitivity for detecting subtle functional networks even from deep brain regions underscores the value of high‐performance coil systems for advancing our understanding of neural architecture in NHPs. The successful demonstration of resting‐state functional connectivity with our system also establishes an important foundation for more complex functional imaging paradigms. The ability to capture intrinsic functional organization at submillimeter resolution suggests that our coil system should similarly support acquisition of task‐evoked responses with high spatial specificity, enabling investigations of cortical and subcortical processing with unprecedented detail.

Our RF coil system was designed to be compatible for both anesthetized and awake experiments with a commonly used sphinx position setup. However, the validation presented here was performed under anesthesia only. In designing the system, we prioritized high channel count and coil complexity while addressing significant mechanical constraints for NHP setups. These two requirements are rarely addressed together in UHF MRI coil designs for NHPs, yet both are essential for neuroscience investigations approximating natural behavior. The custom housing accommodates the sphinx position commonly used in awake setups, while maintaining a clear line of sight for visual stimulus delivery. This open visual field configuration, integrated within a mechanically stable and compact geometry compatible with a specific *B*_0_ shim array, fulfills the dual requirements of visual‐stimulus compatibility and high‐channel performance at UHF.

Looking toward future developments, several promising avenues will further enhance this RF coil system's capabilities. First, we limited the dipole number of channels to 6 due to space constraints and potential high Tx‐coupling with adjacent dipoles. Expanding the dipole array to eight elements with appropriate spacing would enhance deeper structure coverage without sacrificing performance in other regions. The persistent challenge of weak excitation in posterior brain regions likely stems from both geometric constraints related to the awake NHP restraint box and head positioning along the *z*‐axis. To address this limitation, we are exploring dynamic transmit techniques that could improve coverage through adaptive phase adjustments. In this work, phase‐only shimming was used for consistency with electromagnetic simulations; however, RF shimming using combined amplitude and phase optimization can be implemented without added operational complexity and could improve *B*_1_^+^ homogeneity in posterior brain regions (Figure S2, middle row). Approaches such as kT‐points [63] or multi‐channel transmit shimming with universal pulses [71] are particularly promising directions to further enhance *B*_1_^+^ homogeneity (Figure S2, bottom row). Implementing such techniques in pTX mode would address current excitation limitations while enabling dynamic adaptations to accommodate different head positions or shapes, significantly enhancing the system's versatility for diverse experimental needs. Second, increasing the number of channels of the loop array will enhance cortical coverage, especially in superior and posterior brain regions that remain undercovered by our current configuration. Additional loop elements in these locations could improve sensitivity in these areas while maintaining the compact geometry required for visual access. Additionally, a limitation of the present study is that all experimental validations were conducted under anesthesia. Although the coil was mechanically designed to be compatible with awake NHP imaging setups, validation under awake conditions represents an important next step. Finally, the demonstrated capability to obtain high‐quality resting‐state functional connectivity patterns at 0.75 mm isotropic resolution establishes a foundation for more challenging acquisitions, including task‐evoked responses with multiple experimental conditions and paradigms. Future work will also focus on implementing such protocols, ultimately enabling comprehensive investigations of neural function across diverse behavioral states.

## Funding

This work was supported by the French Alternative Energies and Atomic Energy Commission, the exploratory program; Agence Nationale de la Recherche (ANR‐20‐CE37‐0005); Fondation Leducq (NEUROVASC7T).

## Supporting information


**Table S1.** Bench characterization of the 16‐channel receive loop array. Measured S11 matching, preamplifier decoupling (preamplifier connected vs. 50 Ω termination), and active detuning performance (detuned vs. 50 Ω) are reported for all loop elements.
**Table S2.** Bench characterization of the 6‐channel transceive dipole array. Measured S11 matching and preamplifier decoupling (preamplifier connected vs. 50 Ω termination) are reported for all dipole elements.
**Figure S1.** Three noise correlation matrices of the 22 receive elements for different sessions and animals. Dot lines separate the 6 dipoles and the 16 loops.
**Figure S2.** Simulated flip‐angle maps from in vivo individual‐channel B1+ maps using different transmit optimization strategies targeting a flip angle of 10°. For each strategy, the mean, standard deviation, and relative standard deviation (RSD) of the flip angle within the brain mask are reported.

## Data Availability

The data that support the findings of this study are available from the corresponding author upon reasonable request. Raw data and code used in the present study can be made available from the corresponding author upon request.

## References

[1] B. Gruber , J. P. Stockmann , A. Mareyam , et al., “A 128‐Channel Receive Array for Cortical Brain Imaging at 7 T,” Magnetic Resonance in Medicine 90, no. 6 (2023): 2592–2607, 10.1002/mrm.29798. 37582214 PMC10543549

[2] P. F. Gapais , M. Luong , E. Giacomini , et al., “A 32‐Channel High‐Impedance Honeycomb‐Shaped Receive Array for Temporal Lobes Exploration at 11.7T,” Magnetic Resonance in Medicine 93, no. 1 (2025): 433–447, 10.1002/mrm.30274. 39219305

[3] N. I. Avdievich , A. V. Nikulin , L. Ruhm , et al., “A 32‐Element Loop/Dipole Hybrid Array for Human Head Imaging at 7 T,” Magnetic Resonance in Medicine 88, no. 4 (2022): 1912–1926, 10.1002/mrm.29347. 35766426

[4] M. F. G. Luong and E. Chazel , “A Compact 16Tx‐32Rx Geometrically Decoupled Phased Array for 11.7 T MRI,” in Proceedings of the 31st Annual Meeting of the International Society of Magnetic Resonance in Medicine (London, UK) (ISMRM, 2022), 707.

[5] P. F. Gapais , M. Luong , F. Nizery , et al., “Efficiently Building Receive Arrays With Electromagnetic Simulations and Additive Manufacturing: A Two‐Layer, 32‐Channel Prototype for 7T Brain MRI,” Magnetic Resonance in Medicine 91, no. 3 (2024): 1254–1267, 10.1002/mrm.29931. 37986237

[6] R. L. Lagore , S. Moeller , J. Zimmermann , et al., “An 8 Dipole Transceive and 24 Loop Receive Array for Non‐Human Primate Head Imaging at 10.5T,” NMR in Biomedicine 34, no. 4 (2021): e4472, 10.1002/nbm.4472. 33511726 PMC8103796

[7] A. A. Bhosale , Y. Zhao , and X. Zhang , “Electric Field and SAR Reduction in High‐Impedance RF Arrays by Using High Permittivity Materials for 7T MR Imaging,” PLoS One 19, no. 7 (2024): e0305464, 10.1371/journal.pone.0305464. 38959266 PMC11221758

[8] P. F. de Van Moortele , C. Akgun , G. Adriany , et al., “B1 Destructive Interferences and Spatial Phase Patterns at 7 T With a Head Transceiver Array Coil,” Magnetic Resonance in Medicine 54, no. 6 (2005): 1503–1518, 10.1002/mrm.20708. 16270333

[9] C. J. Donahue , M. F. Glasser , T. M. Preuss , J. K. Rilling , and D. C. Van Essen , “Quantitative Assessment of Prefrontal Cortex in Humans Relative to Nonhuman Primates,” Proceedings of the National Academy of Sciences of the United States of America 115, no. 22 (2018): E5183–E5192, 10.1073/pnas.1721653115. 29739891 PMC5984508

[10] M. F. Glasser , T. S. Coalson , E. C. Robinson , et al., “A Multi‐Modal Parcellation of Human Cerebral Cortex,” Nature 536, no. 7615 (2016): 171–178, 10.1038/nature18933. 27437579 PMC4990127

[11] X. Li , Q. Zhu , T. Janssens , J. T. Arsenault , and W. Vanduffel , “In Vivo Identification of Thick, Thin, and Pale Stripes of Macaque Area V2 Using Submillimeter Resolution (f)MRI at 3 T,” Cerebral Cortex 29, no. 2 (2019): 544–560, 10.1093/cercor/bhx337. 29300915

[12] Z. Quan , Y. Gao , S. Qu , et al., “A 16‐Channel Loop Array for In Vivo Macaque Whole‐Brain Imaging at 3T,” Magnetic Resonance Imaging 68 (2020): 167–172, 10.1016/j.mri.2020.02.008. 32081631 PMC7784245

[13] T. Janssens , B. Keil , P. Serano , et al., “A 22‐Channel Receive Array With Helmholtz Transmit Coil for Anesthetized Macaque MRI at 3 T,” NMR in Biomedicine 26, no. 11 (2013): 1431–1440, 10.1002/nbm.2970. 23703859

[14] J. A. Autio , M. F. Glasser , T. Ose , et al., “Towards HCP‐Style Macaque Connectomes: 24‐Channel 3T Multi‐Array Coil, MRI Sequences and Preprocessing,” NeuroImage 215 (2020): 116800, 10.1016/j.neuroimage.2020.116800. 32276072 PMC7116593

[15] F. Lou , X. Tang , Z. Quan , et al., “A 16‐Channel Loop Array for In Vivo Macaque Whole‐Brain Imaging at 7 T,” Magnetic Resonance Imaging 102 (2023): 179–183, 10.1016/j.mri.2023.06.014. 37356599

[16] Y. Gao , A. Mareyam , Y. Sun , et al., “A 16‐Channel AC/DC Array Coil for Anesthetized Monkey Whole‐Brain Imaging at 7T,” NeuroImage 207 (2020): 116396, 10.1016/j.neuroimage.2019.116396. 31778818 PMC7309650

[17] K. M. Gilbert , J. S. Gati , K. Barker , S. Everling , and R. S. Menon , “Optimized Parallel Transmit and Receive Radiofrequency Coil for Ultrahigh‐Field MRI of Monkeys,” NeuroImage 125 (2016): 153–161, 10.1016/j.neuroimage.2015.10.048. 26497267

[18] E. Yacoub , M. D. Grier , E. J. Auerbach , et al., “Ultra‐High Field (10.5 T) Resting State fMRI in the Macaque,” NeuroImage 223 (2020): 117349, 10.1016/j.neuroimage.2020.117349. 32898683 PMC7745777

[19] A. G. Xu , M. Qian , F. Tian , et al., “Focal Infrared Neural Stimulation With High‐Field Functional MRI: A Rapid Way to Map Mesoscale Brain Connectomes,” Science Advances 5, no. 4 (2019): eaau7046, 10.1126/sciadv.aau7046. 31032400 PMC6482007

[20] T. L. Wu , P. F. Yang , F. Wang , et al., “Intrinsic Functional Architecture of the Non‐Human Primate Spinal Cord Derived From fMRI and Electrophysiology,” Nature Communications 10, no. 1 (2019): 1416, 10.1038/s41467-019-09485-3. PMC6440970 30926817

[21] G. Chen , F. Wang , J. C. Gore , and A. W. Roe , “Layer‐Specific BOLD Activation in Awake Monkey V1 Revealed by Ultra‐High Spatial Resolution Functional Magnetic Resonance Imaging,” NeuroImage 64 (2013): 147–155, 10.1016/j.neuroimage.2012.08.060. 22960152 PMC3508288

[22] L. M. Chen , G. H. Turner , R. M. Friedman , et al., “High‐Resolution Maps of Real and Illusory Tactile Activation in Primary Somatosensory Cortex in Individual Monkeys With Functional Magnetic Resonance Imaging and Optical Imaging,” Journal of Neuroscience 27, no. 34 (2007): 9181–9191, 10.1523/JNEUROSCI.1588-07.2007. 17715354 PMC6672200

[23] N. K. Logothetis , J. Pauls , M. Augath , T. Trinath , and A. Oeltermann , “Neurophysiological Investigation of the Basis of the fMRI Signal,” Nature 412 (2001): 150–157. 11449264 10.1038/35084005

[24] M. Qian , J. Wang , Y. Gao , et al., “Multiple Loci for Foveolar Vision in Macaque Monkey Visual Cortex,” Nature Neuroscience 28, no. 1 (2025): 137–149, 10.1038/s41593-024-01810-4. 39639181 PMC11706779

[25] W. Vanduffel , D. Fize , J. B. Mandeville , et al., “Visual Motion Processing Investigated Using Contrast Agent‐Enhanced fMRI in Awake Behaving Monkeys,” Neuron 32, no. 4 (2001): 565–577, 10.1016/s0896-6273(01)00502-5. 11719199

[26] C. H. Choi , A. Webb , S. Orzada , M. Kelenjeridze , N. J. Shah , and J. Felder , “A Review of Parallel Transmit Arrays for Ultra‐High Field MR Imaging,” IEEE Reviews in Biomedical Engineering 17 (2023): 1–19, 10.1109/RBME.2023.3244132. 37022919

[27] S. M. Hong , J. H. Park , M. K. Woo , Y. B. Kim , and Z. H. Cho , “New Design Concept of Monopole Antenna Array for UHF 7T MRI,” Magnetic Resonance in Medicine 71, no. 5 (2014): 1944–1952, 10.1002/mrm.24844. 23818275

[28] A. J. E. Raaijmakers , P. R. Luijten , and C. van den Berg , “Dipole Antennas for Ultrahigh‐Field Body Imaging: A Comparison With Loop Coils,” NMR in Biomedicine 29, no. 9 (2016): 1122–1130, 10.1002/nbm.3356. 26278544

[29] J. D. Clément , R. Gruetter , and Ö. Ipek , “A Human Cerebral and Cerebellar 8‐Channel Transceive RF Dipole Coil Array at 7T,” Magnetic Resonance in Medicine 81, no. 2 (2019): 1447–1458, 10.1002/mrm.27476. 30226637

[30] X. Yan , J. C. Gore , and W. A. Grissom , “Self‐Decoupled Radiofrequency Coils for Magnetic Resonance Imaging,” Nature Communications 9, no. 1 (2018): 3481, 10.1038/s41467-018-05585-8. PMC6113296 30154408

[31] M. A. Griswold , P. M. Jakob , R. M. Heidemann , et al., “Generalized Autocalibrating Partially Parallel Acquisitions (GRAPPA),” Magnetic Resonance in Medicine 47, no. 6 (2002): 1202–1210, 10.1002/mrm.10171. 12111967

[32] K. P. Pruessmann , M. Weiger , M. B. Scheidegger , and P. Boesiger , “SENSE: Sensitivity Encoding for Fast MRI,” Magnetic Resonance in Medicine 42, no. 5 (1999): 952–962, 10.1002/(SICI)1522-2594(199911)42:5<952::AID-MRM16>3.0.CO;2-S. 10542355

[33] K. Uğurbil , E. Auerbach , S. Moeller , et al., “Brain Imaging With Improved Acceleration and SNR at 7 Tesla Obtained With 64‐Channel Receive Array,” Magnetic Resonance in Medicine 82, no. 1 (2019): 495–509, 10.1002/mrm.27695. 30803023 PMC6491243

[34] B. Keil and L. L. Wald , “Massively Parallel MRI Detector Arrays,” Journal of Magnetic Resonance 229 (2013): 75–89, 10.1016/j.jmr.2013.02.001. 23453758 PMC3740730

[35] A. Reykowski , S. M. Wright , and J. R. Porter , “Design of Matching Networks for Low Noise Preamplifiers,” Magnetic Resonance in Medicine 33, no. 6 (1995): 848–852, 10.1002/mrm.1910330617. 7651124

[36] P. B. Roemer , W. A. Edelstein , C. E. Hayes , S. P. Souza , and O. M. Mueller , “The NMR Phased Array,” Magnetic Resonance in Medicine 16, no. 2 (1990): 192–225, 10.1002/mrm.1910160203. 2266841

[37] W. Wang , V. Zhurbenko , J. D. Sánchez‐Heredia , and J. H. Ardenkjaer‐Larsen , “Three‐Element Matching Networks for Receive‐Only MRI Coil Decoupling,” Magnetic Resonance in Medicine 85, no. 1 (2021): 544–550, 10.1002/mrm.28416. 32686177

[38] C. Ianniello , J. A. de Zwart , Q. Duan , et al., “Synthesized Tissue‐Equivalent Dielectric Phantoms Using Salt and Polyvinylpyrrolidone Solutions,” Magnetic Resonance in Medicine 80, no. 1 (2018): 413–419, 10.1002/mrm.27005. 29159985 PMC5876111

[39] M. Froesel , K. Ikuchi , Q. Zhu , et al., “High‐Resolution fMRI Reveals a Dorsal Brain Pathway Selective for Conspecific Vocalizations in Macaques,” Imaging Neuroscience 3 (2025): IMAG.a.108, 10.1162/IMAG.a.108. 40821761 PMC12351310

[40] B. R. Cottereau , A. T. Smith , S. Rima , et al., “Processing of Egomotion‐Consistent Optic Flow in the Rhesus Macaque Cortex,” Cerebral Cortex 27, no. 1 (2017): 330–343, 10.1093/cercor/bhw412. 28108489 PMC5939222

[41] E. Djaballah , A. Uthayakumar , M. Bouchet , et al., “A 48‐Channel Multi‐Coil Shim Array for B0 Inhomogeneities Correction in the NHP Brain at 7T,” in Proceedings of the International Society for Magnetic Resonance in Medicine, vol. 32 (ISMRM, 2024).

[42] L. Darrasse and G. Kassab , “Quick Measurement of NMR‐Coil Sensitivity With a Dual‐Loop Probe,” Review of Scientific Instruments 64, no. 7 (1993): 1841–1844, 10.1063/1.1144020.

[43] A. Amadon , F. Mauconduit , A. Vignaud , and N. Boulant , Slice Profile Corrections in the XFL (Magnetization‐Prepared Turbo‐FLASH) B1‐Mapping Sequence (ISMRM, 2015), https://archive.ismrm.org/2015/2377.html.

[44] D. O. Brunner and K. P. Pruessmann , “B Interferometry for the Calibration of RF Transmitter Arrays,” Magnetic Resonance in Medicine 61, no. 6 (2009): 1480–1488, 10.1002/mrm.21893. 19353666

[45] H. P. Fautz , M. Vogel , P. Gross , A. Kerr , and Y. Zhu , “B1 Mapping of Coil Arrays for Parallel Transmission,” 2008.

[46] S. Moeller , E. Yacoub , C. A. Olman , et al., “Multiband Multislice GE‐EPI at 7 Tesla, With 16‐Fold Acceleration Using Partial Parallel Imaging With Application to High Spatial and Temporal Whole‐Brain fMRI,” Magnetic Resonance in Medicine 63, no. 5 (2010): 1144–1153, 10.1002/mrm.22361. 20432285 PMC2906244

[47] P. Kellman and E. R. McVeigh , “Image Reconstruction in SNR Units: A General Method for SNR Measurement,” Magnetic Resonance in Medicine 54, no. 6 (2005): 1439–1447, 10.1002/mrm.20713. 16261576 PMC2570032

[48] M. Jenkinson , C. F. Beckmann , T. E. J. Behrens , M. W. Woolrich , and S. M. Smith , “FSL,” NeuroImage 62, no. 2 (2012): 782–790, 10.1016/J.NEUROIMAGE.2011.09.015. 21979382

[49] N. J. Tustison , P. A. Cook , A. J. Holbrook , et al., “The ANTsX Ecosystem for Quantitative Biological and Medical Imaging,” Scientific Reports 11, no. 1 (2021): 1–13, 10.1038/s41598-021-87564-6. 33907199 PMC8079440

[50] R. W. Cox , “AFNI: Software for Analysis and Visualization of Functional Magnetic Resonance Neuroimages,” Computers and Biomedical Research 29, no. 3 (1996): 162–173, 10.1006/cbmr.1996.0014. 8812068

[51] B. Fischl , “FreeSurfer,” NeuroImage 62, no. 2 (2012): 774–781, 10.1016/j.neuroimage.2012.01.021. 22248573 PMC3685476

[52] K. Gorgolewski , C. D. Burns , C. Madison , et al., “Nipype: A Flexible, Lightweight and Extensible Neuroimaging Data Processing Framework in Python,” Frontiers in Neuroinformatics 5 (2011): 13, 10.3389/fninf.2011.00013. 21897815 PMC3159964

[53] F. Pedregosa , G. Varoquaux , A. Gramfort , et al., “Scikit‐Learn: Machine Learning in Python,” Journal of Machine Learning Research 12, no. 85 (2011): 2825–2830.

[54] S. Moeller , P. K. Pisharady , S. Ramanna , et al., “NOise Reduction With DIstribution Corrected (NORDIC) PCA in dMRI With Complex‐Valued Parameter‐Free Locally Low‐Rank Processing,” NeuroImage 226 (2021): 117539, 10.1016/j.neuroimage.2020.117539. 33186723 PMC7881933

[55] P. F. Balan , Q. Zhu , X. Li , et al., “MEBRAINS 1.0: A New Population‐Based Macaque Atlas,” Imaging Neuroscience 2 (2024): 1–26, 10.1162/imag_a_00077. PMC12235559 40800436

[56] K. J. Friston , A. P. Holmes , K. J. Worsley , J. P. Poline , C. D. Frith , and R. S. J. Frackowiak , “Statistical Parametric Maps in Functional Imaging: A General Linear Approach,” Human Brain Mapping 2, no. 4 (1994): 189–210, 10.1002/hbm.460020402.

[57] M. A. Lindquist , S. Geuter , T. D. Wager , and B. S. Caffo , “Modular Preprocessing Pipelines Can Reintroduce Artifacts Into fMRI Data,” Human Brain Mapping 40, no. 8 (2019): 407676, 10.1101/407676. PMC6865661 30666750

[58] Y. Behzadi , K. Restom , J. Liau , and T. T. Liu , “A Component Based Noise Correction Method (CompCor) for BOLD and Perfusion Based fMRI,” NeuroImage 37, no. 1 (2007): 90–101, 10.1016/j.neuroimage.2007.04.042. 17560126 PMC2214855

[59] M. N. Hallquist , K. Hwang , and B. Luna , “The Nuisance of Nuisance Regression: Spectral Misspecification in a Common Approach to Resting‐State fMRI Preprocessing Reintroduces Noise and Obscures Functional Connectivity,” NeuroImage 82 (2013): 208–225, 10.1016/j.neuroimage.2013.05.116. 23747457 PMC3759585

[60] Q. Zhu , J. Zhang , Y. L. L. Luo , D. D. Dilks , and J. Liu , “Resting‐State Neural Activity Across Face‐Selective Cortical Regions Is Behaviorally Relevant,” Journal of Neuroscience 31, no. 28 (2011): 10323–10330, 10.1523/JNEUROSCI.0873-11.2011. 21753009 PMC6623054

[61] Q. Zhu and W. Vanduffel , “Submillimeter fMRI Reveals a Layout of Dorsal Visual Cortex in Macaques, Remarkably Similar to New World Monkeys,” National Academy of Sciences of the United States of America 116, no. 6 (2019): 2306–2311, 10.1073/pnas.1805561116. PMC6369784 30674668

[62] B. Jung , P. A. Taylor , J. Seidlitz , et al., “A Comprehensive Macaque fMRI Pipeline and Hierarchical Atlas,” NeuroImage 235 (2021), 10.1101/2020.08.05.237818. PMC9272767 33789138

[63] M. A. Cloos , N. Boulant , M. Luong , et al., “kT‐Points: Short Three‐Dimensional Tailored RF Pulses for Flip‐Angle Homogenization Over an Extended Volume,” Magnetic Resonance in Medicine 67, no. 1 (2012): 72–80, 10.1002/mrm.22978. 21590724

[64] J. L. Vincent , G. H. Patel , M. D. Fox , et al., “Intrinsic Functional Architecture in the Anaesthetized Monkey Brain,” Nature 447, no. 7140 (2007): 83–86, 10.1038/nature05758. 17476267

[65] D. S. Margulies , J. L. Vincent , C. Kelly , et al., “Precuneus Shares Intrinsic Functional Architecture in Humans and Monkeys,” National Academy of Sciences of the United States of America 106, no. 47 (2009): 20069–20074, 10.1073/pnas.0905314106. PMC2775700 19903877

[66] J. L. Vincent , I. Kahn , D. C. Van Essen , and R. L. Buckner , “Functional Connectivity of the Macaque Posterior Parahippocampal Cortex,” Journal of Neurophysiology 103, no. 2 (2010): 793–800, 10.1152/JN.00546.2009/ASSET/IMAGES/LARGE/Z9K0021099210005.JPEG. 19955295 PMC2822694

[67] M. K. Woo , L. DelaBarre , M. Waks , et al., “A 16‐Channel Dipole Antenna Array for Human Head Magnetic Resonance Imaging at 10.5 Tesla,” Sensors 21, no. 21 (2021): 7250, 10.3390/s21217250. 34770558 PMC8587099

[68] R. M. Hutchison , L. S. Leung , S. M. Mirsattari , J. S. Gati , R. S. Menon , and S. Everling , “Resting‐State Networks in the Macaque at 7 T,” NeuroImage 56, no. 3 (2011): 1546–1555, 10.1016/j.neuroimage.2011.02.063. 21356313

[69] C. M. Garin , Y. Hori , S. Everling , et al., “An Evolutionary Gap in Primate Default Mode Network Organization,” Cell Reports 39, no. 2 (2022): 110669, 10.1016/j.celrep.2022.110669. 35417698 PMC9088817

[70] T. Teichert , J. Grinband , J. Hirsch , and V. P. Ferrera , “Effects of Heartbeat and Respiration on Macaque fMRI: Implications for Functional Connectivity,” Neuropsychologia 48, no. 7 (2010): 1886–1894, 10.1016/j.neuropsychologia.2009.11.026. 19969009 PMC2876227

[71] V. Gras , A. Vignaud , A. Amadon , D. Le Bihan , and N. Boulant , “Universal Pulses: A New Concept for Calibration‐Free Parallel Transmission,” Magnetic Resonance in Medicine 77, no. 2 (2017): 635–643, 10.1002/mrm.26148. 26888654

