# Supplementary material for: A Hybrid RF Coil for Whole‐Brain Imaging in Non‐Human Primates at 7 T
Source: Magn Reson Med. 2026 Mar 24;96(2):996–1010. doi: 10.1002/mrm.70361 (PMC13269185; doi:10.1002/mrm.70361)
Supplement: Supplementary file 1 — Table S1. Bench characterization of the 16‐channel receive loop array. Measured S11 matching, preamplifier decoupling (preamplifier connected vs. 50 Ω termination), and active detuning performance (detuned vs. 50 Ω) are reported for all loop elements. Table S2. Bench characterization of the 6‐channel transceive dipole array. Measured S11 matching and preamplifier decoupling (preamplifier connected vs. 50 Ω termination) are reported for all dipole elements. Figure S1. Three noise correlation matrices of the 22 receive elements for different sessions and animals. Dot lines separate the 6 dipoles and the 16 loops. Figure S2. Simulated flip‐angle maps from in vivo individual‐channel B1+ maps using different transmit optimization strategies targeting a flip angle of 10°. For each strategy, the mean, standard deviation, and relative standard deviation (RSD) of the flip angle within the brain mask are reported. [file MRM-96-996-s001.docx]

# A Hybrid RF Coil for Whole-Brain Imaging in Non-Human Primates at 7T

SUPPORTING INFORMATION

Authors: Elias Djaballah^1,2^, Éric Giacomini^2^, Paul-François Gapais^2^, Michel Luong^3^, Alexis Amadon^2^ and Qi Zhu^1^*****

^1^Cognitive Neuroimaging Unit, INSERM, CEA, Université Paris-Saclay, NeuroSpin Center, GIF-SUR-YVETTE, France

^2^BAOBAB, Université Paris-Saclay, CEA/Joliot/NeuroSpin, GIF-SUR-YVETTE, France

^3^Université Paris-Saclay, CEA/DRF/IRFU/DACM, GIF-SUR-YVETTE, France

*****Corresponding author: Qi Zhu (qi.zhu@cea.fr)

| **Element** | **S11 (dB)** | **Preamp decoupling (Preamp vs. 50 Ω) (dB)** | **Detuning (Detuned vs. 50 Ω) (dB)** |
| --- | --- | --- | --- |
| L1 | −18.9 | 19.4 | 35.6 |
| L2 | −20.6 | 18.6 | 38.4 |
| L3 | −15.7 | 18.3 | 37.5 |
| L4 | −19.4 | 21.1 | 36.5 |
| L5 | −14.2 | 20.5 | 40.1 |
| L6 | −16.2 | 19.6 | 41.4 |
| L7 | −19.5 | 17.9 | 37.7 |
| L8 | −19.2 | 24.1 | 34.3 |
| R1 | −17.8 | 18.8 | 36.8 |
| R2 | −22.1 | 20.3 | 33.3 |
| R3 | −16.9 | 16.8 | 41.2 |
| R4 | −22.3 | 20.2 | 39.5 |
| R5 | −18.2 | 19.3 | 35.1 |
| R6 | −20.4 | 20.2 | 34.4 |
| R7 | −23.6 | 19.6 | 38.3 |
| R8 | -18.9 | 18.5 | 35.9 |

**Table S1.** Bench characterization of the 16-channel receive loop array. Measured S11 matching, preamplifier decoupling (preamplifier connected vs. 50 Ω termination), and active detuning performance (detuned vs. 50 Ω) are reported for all loop elements.

| **Element** | **S11 (dB)** | **Preamp decoupling (Preamp vs. 50 Ω) (dB)** |
| --- | --- | --- |
| D1 | −13.8 | 22.3 |
| D2 | −11.4 | 21.9 |
| D3 | −10.6 | 22.1 |
| D4 | −10.2 | 20.9 |
| D5 | −12.7 | 21.6 |
| D6 | −13.2 | 22.2 |

**Table S2.** Bench characterization of the 6-channel transceive dipole array. Measured S11 matching and preamplifier decoupling (preamplifier connected vs. 50 Ω termination) are reported for all dipole elements.

**Figure S1.** Three noise correlation matrices of the 22 receive elements for different sessions and animals. Dot lines separate the 6 dipoles and the 16 loops.

**Figure S2.** Simulated flip-angle maps from in vivo individual-channel B1+ maps using different transmit optimization strategies targeting a flip angle of 10°. For each strategy, the mean, standard deviation, and relative standard deviation (RSD) of the flip angle within the brain mask are reported.
